# Meta-analysis of modified Stoppa approach and ilioinguinal approach in anterior pelvic ring and acetabular fractures

**DOI:** 10.1097/MD.0000000000018395

**Published:** 2020-01-24

**Authors:** Hao Wu, LiJun Zhang, XiaoMin Guo, XiaoJun Jiang

**Affiliations:** a Department of Orthopedics, Sihong People's Hospital, Suqian, Jiangsu; b Department of Orthopedics, The 5th People's Hospital of Jinan, Jinan, Shandong; c Chuxiong Medical College, Chuxiong, Yunnan; d Department of Orthopedics, Wujin People's Hospital, Changzhou, Jiangsu, China.

**Keywords:** mini-incision, operative approach, pelvic fracture, review

## Abstract

**Background:**

The purpose of this meta-analysis was to compare the efficacy of the modified Stoppa approach (MSA) and ilioinguinal approach (IA) in the treatment of anterior pelvic ring and acetabular fractures.

**Methods:**

A literature search was conducted using PubMed, Embase, and Cochrane database for articles that compared MSA and IA in the treatment of anterior pelvic ring and acetabular fractures. All the included articles were evaluated by 2 trained reviewers in accordance with the Cochrane Collaboration Handbook for potential risk. The Jadad decision algorithm and Downs and Black scores were also used to assess the quality of the included studies. The extracted data included operative time, intraoperative blood loss, reduction quality, clinical outcome, and complications.

**Results:**

Five articles were included in this meta-analysis, with 186 patients in the MSA group and 219 patients in the IA group. Compared with IA, MSA significantly shortened the operative time (*P* = .0002), decreased intraoperative blood loss (*P* = .002), and provided better reduction quality (*P* = .03). Meanwhile, this meta-analysis suggests no significant difference between MSA and IA regarding clinical outcomes (*P* = .63) and complications (*P* = .34). The subgroup analysis of complications also showed no statistically significant difference between the 2 groups (including infection, and vascular and nerve injuries).

**Conclusion:**

According to this meta-analysis, the currently available evidence suggests that MSA can significantly shorten operative time, decrease intraoperative blood loss, and provide better reduction quality than IA in the treatment of anterior pelvic ring and acetabular fractures. In addition, in terms of clinical outcomes and complications, no significant differences were found between the 2 groups.

**Level of Evidence:** Level IV, meta-analysis.

## 1 Introduction

Acetabular fractures are one of the most difficult fractures to treat because of the complexity of anatomical surgery, and is considered one of the most challenging operations for orthopedic surgeons.^[1]^ Anatomical reduction of fractures and joint reconstruction are the basis for the treatment of acetabular fractures, which have been recognized by most orthopedic surgeons.^[2]^ Choosing the appropriate surgical approach for the treatment of anterior pelvic ring and acetabular fractures is the key to achieving an anatomical reduction of fractures and reducing complications.^[2,3]^

**Figure 1:**
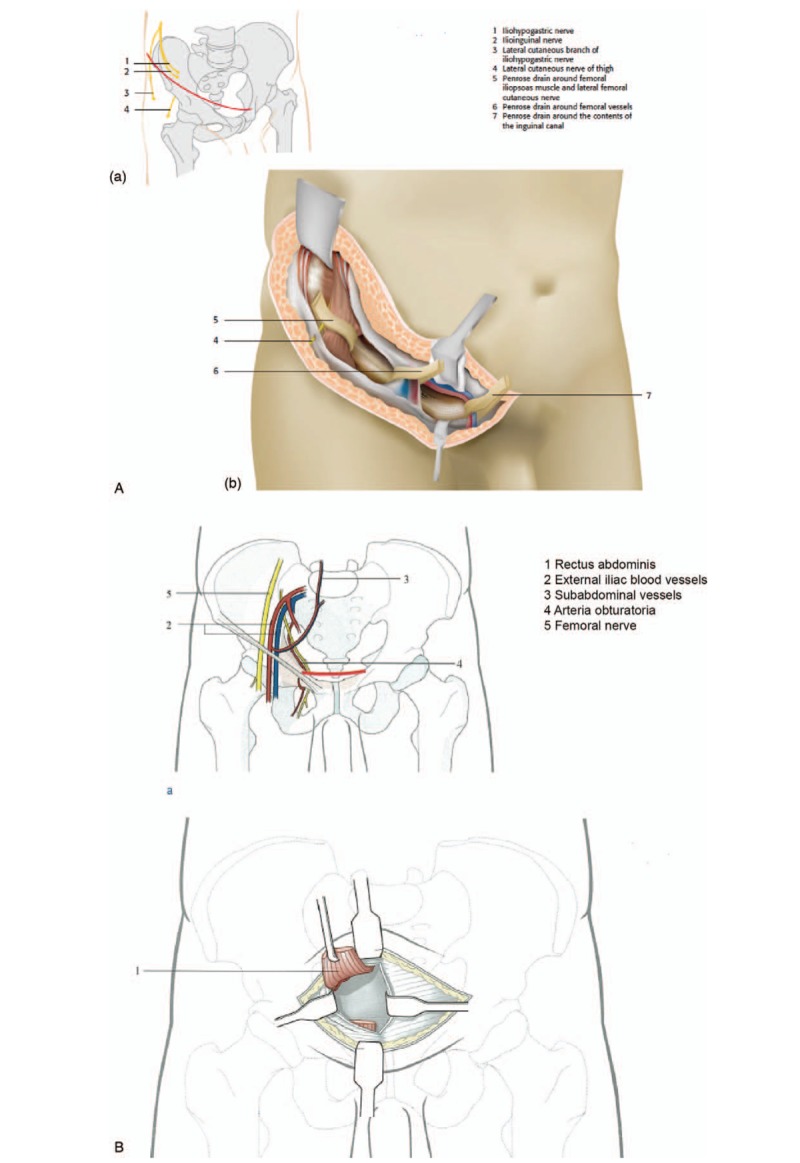
. The schematic diagram of (A) ilioinguinal approach and (B) modified Stoppa approach.

Ever since Letournel^[4]^ proposed the ilioinguinal approach (IA), it has been widely used to treat pelvic ring and acetabular fractures. IA approach (Fig. 1) can provide many advantages such as good exposure of acetabular fracture, no separate abductor muscle, low sciatic nerve injury rate, easy-to-hide postoperative scar, and rapid recovery.^[5]^ The anatomical reduction rate was reported to reach 45% to 74%.^[6,7]^ However, the approach requires repeated traction of the lateral femoral cutaneous nerve, femoral nerve, and extra-orbital blood vessels during surgery, which may lead to complications such as nerve palsy, vasospasm, and venous embolism.^[2,7,8]^ In 1993, Hirvensalo et al^[9]^ first reported the modified Stoppa approach (MSA) to treat pelvic ring or acetabular fractures. MSA (Fig. 1) can decrease surgical trauma, provide good visualization, and make reduction and fixation of medially displaced fractures easier.^[10–13]^ The anatomical reduction rates were reported to range from 59% to 82%.^[10,11,13]^ MSA was reported to be useful for all pelvic fractures suitable for IA treatment.^[14]^

Recently, some scholars compared the efficacy of the 2 approaches in the treatment of anterior pelvic ring and acetabular fractures,^[12,14–17]^ but few scholars have conducted a systematic review or meta-analysis to compare the efficacy of the 2 approaches. Therefore, the purpose of this study was to perform a meta-analysis to compare clinical outcomes between MSA and IA for the management of anterior pelvic ring and acetabular fractures.

## 2 Methods

### 2.1 Study design

A literature search was conducted using PubMed, Embase, and Cochrane databases to perform a meta-analysis according to the Preferred Reporting Items for Systematic Reviews and Meta-Analysis statement.^[18]^ This protocol has been registered in PROSPERO. The search strategy was as follows: (((Pelvic fracture [Title/Abstract]) OR acetabulum fracture [Title/Abstract])) AND ((Stoppa approach [Title/Abstract]) OR ilioinguinal approach [Title/Abstract]). The latest article search of this meta-analysis was up to May 10, 2019. At the same time, reference lists of the published studies were checked to identify any suitable references for inclusion.

### 2.2 Eligibility criteria

The inclusion criteria were as follows:

1. articles that compared the outcomes of MSA with those of IA in the treatment of anterior pelvic ring and acetabular fractures;
2. articles that included at least one of the following measurements: operative time, intraoperative blood loss, reduction quality, clinical outcomes, and complications; and
3. articles written in English.

The exclusion criteria were as follows:

1. articles that did not compare MSA and IA;
2. articles from which data could not be extracted;
3. articles not written in English; and
4. articles that did not include at least one of the following measurements: operative time, intraoperative blood loss, reduction quality, clinical outcomes, and complications.

### 2.3 Quality appraisal

In accordance with the inclusion and exclusion criteria, 2 authors read the titles, abstracts, and full texts of the initial examination documents and screened out the documents that met the evaluation independently. Each included article was evaluated by 2 trained authors in accordance with the Cochrane Collaboration Handbook for potential risk, including selection, performance, detection, attrition, reporting, or other biases. Meanwhile, the Jadad decision algorithm^[19]^ was used to assess the quality of randomized controlled trials, and the Downs and Black scores^[20]^ was used to evaluate the quality of nonrandomized controlled trials. If differences exist, they will be discussed or resolved by the third author after reviewing the article.

### 2.4 Data extraction and statistical analyses

Two trained authors extracted the data from the articles, including the authors, year of publication, basic patient information, number of participants, follow-up time, operative time, intraoperative blood loss, reduction quality, clinical outcomes, and complications. If the 2 authors had a disagreement, the third senior professor made the final decision after reviewing the article carefully.

The collected data were analyzed by using the RevMan 5 (version 5.1.4, Cochrane, London, UK) for meta-analysis. The weighted mean difference was used to assess the continuous variables, and the dichotomous variables were evaluated as odds ratios. The associated 95% confidence intervals (CIs) were calculated for each included study, and the statistically significant difference was set at *P* ≤ .05. *Q* and *I*^2^ statistics were performed to assess the heterogeneity among the included studies. Articles were considered as having no heterogeneity when the *P* value was >.10 and *I*^2^ was <50%. We used a random-effects model to calculate the combined effect size to obtain a more conservative result.

## 3 Results

### 3.1 Literature search results and study characteristics

**Figure 2:**
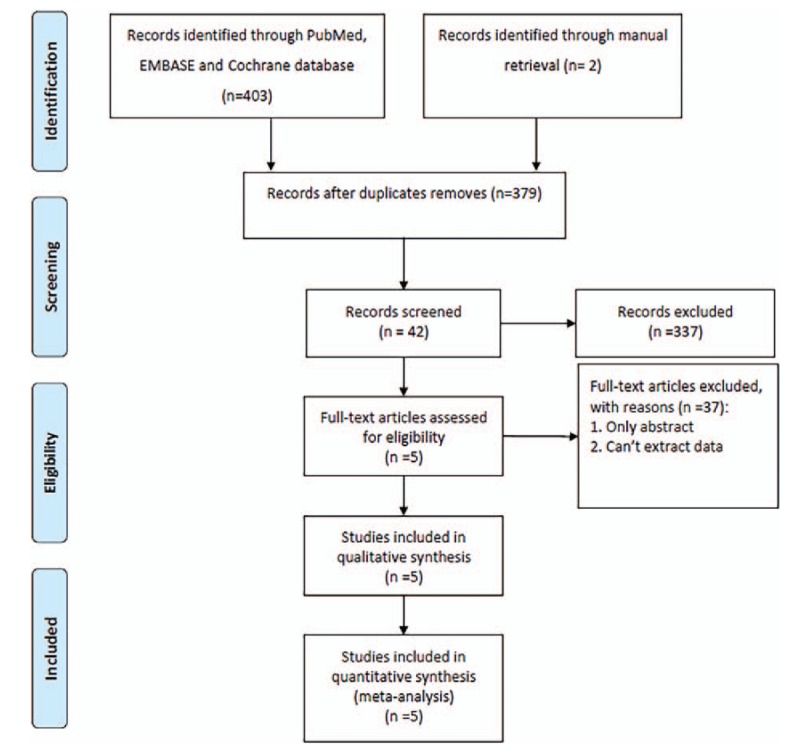
. Preferred Reporting Items for Systematic Reviews and Meta-Analysis flow diagram.

According to the search strategy, 379 articles were identified from the PubMed, Embase, and Cochrane databases, and reference lists of the published studies after duplicate articles were removed; 42 articles remained after the titles and abstracts were screened. Only 5 studies^[12,14–17]^ were included in the meta-analysis after full texts were reviewed. The Preferred Reporting Items for Systematic Reviews and Meta-Analysis flow diagram is illustrated in Figure 2. A total of 405 patients were included in this meta-analysis, with 186 patients in the MSA group and 219 patients in the IA group. Table 1 summarizes the characteristics of each included study. All the included articles had longer than 12-month follow-up.

**Table 1:**
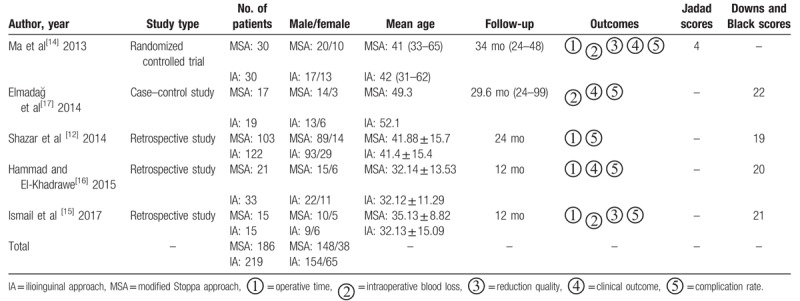
The characteristics of each included study.

### 3.2 Quality assessment

**Figure 3:**
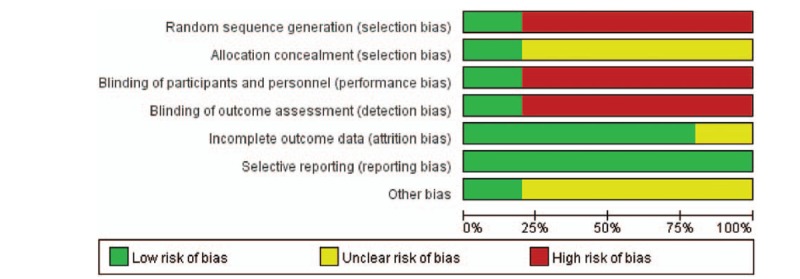
. Risk of bias graph.

**Figure 4:**
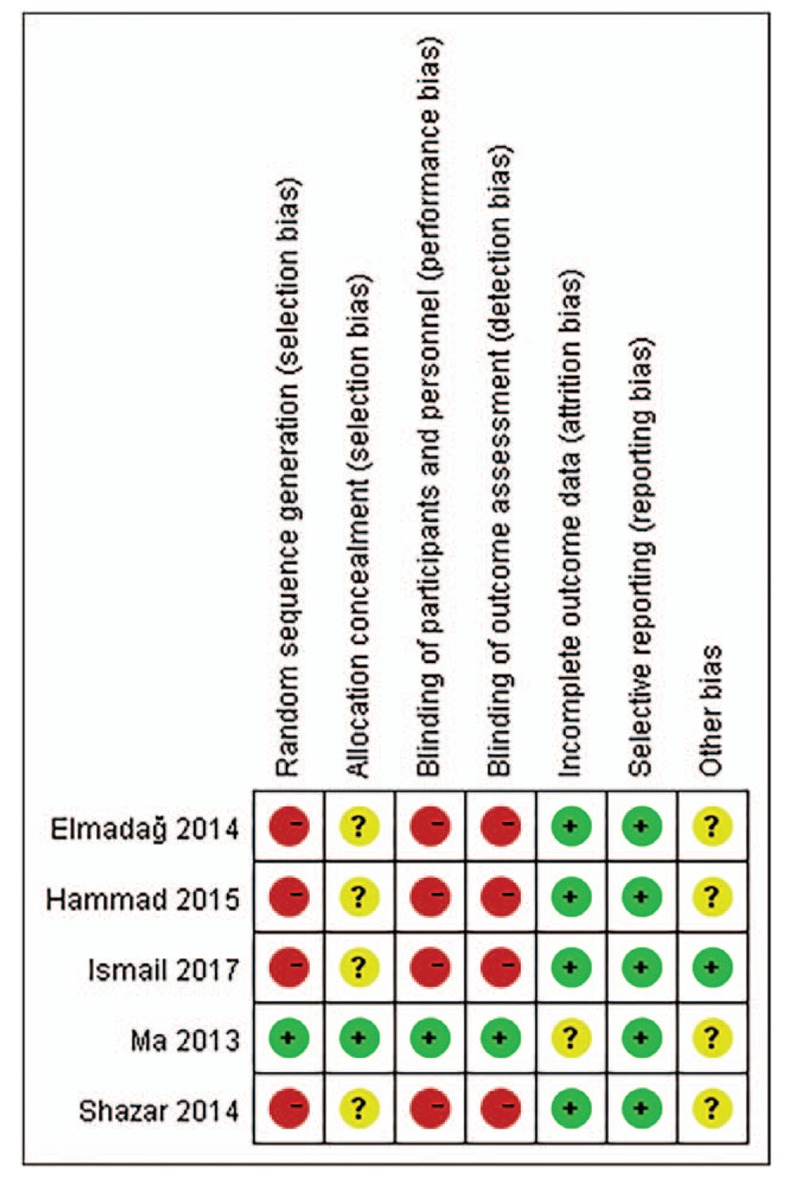
. Risk of bias summary.

Each included study was evaluated by 2 trained reviewers according to the Cochrane Collaboration Handbook for potential risk. The risk of bias graph and risk of bias summary is shown in Figures 3 and 4. The Jadad decision algorithm^[19]^ and Downs and Black scores^[20]^ were also used to assess the quality of the included studies. Ma et al^[14]^ scored 4 points (maximum: 5), and other articles^[12,15–17]^ scored 19 to 22 points (maximum: 30). This means that among these studies, 1 study was of higher quality, and the other articles were of lower quality (Table 1).

### 3.3 Clinical and radiographic outcomes

#### 3.3.1 Operative time

**Figure 5:**
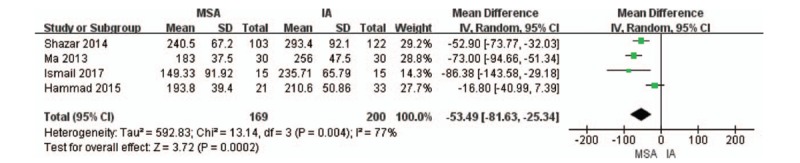
. Forest plots of operative time. CI = confidence interval, IA = ilioinguinal approach, MSA = modified Stoppa approach, SD = standard deviation.

Four articles^[12,14–16]^ reported the operative times between the MSA and IA groups, with 169 patients in the MSA and 200 in the IA. As shown in Figure 5, this meta-analysis suggests that the MSA can statistically shorten the operative time as compared with the IA (*P* = .0002; 95% CI: −81.63 to −25.34).

#### 3.3.2 Intraoperative blood loss

**Figure 6:**
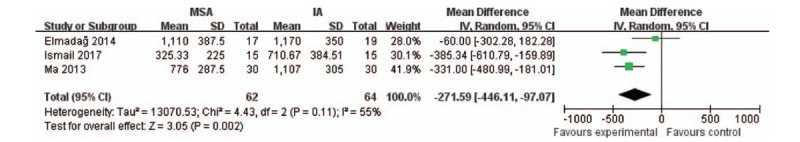
. Forest plots of intraoperative blood loss. CI = confidence interval, IA = ilioinguinal approach, MSA = modified Stoppa approach, SD = standard deviation.

Intraoperative blood loss was assessed in 3 articles,^[14,15,17]^ with 62 patients in the MSA group and 64 patients in the IA group. The meta-analysis revealed a significant difference between the 2 groups in intraoperative blood loss (*P* = .002; 95% CI: −446.11 to −97.07; Fig. 6).

#### 3.3.3 Reduction quality

**Figure 7:**
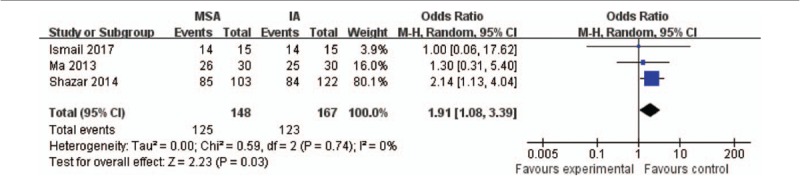
. Forest plots of reduction quality. CI = confidence interval, IA = ilioinguinal approach, MSA = modified Stoppa approach.

Three studies^[12,14,15]^ evaluated the reduction quality in accordance with Matta criteria,^[21]^ including 148 participators in the MSA group and 167 participators in the IA group. Anatomical (<2 mm of displacement) and satisfactory outcomes (2–3 mm) were considered an excellent quality of reduction. Figure 7 suggests that MSA could attain a better quality of reduction than IA (*P* = .03; 95% CI: 1.08–3.39).

#### 3.3.4 Clinical outcome

**Figure 8:**
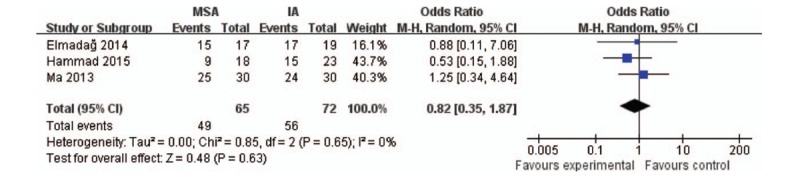
. Forest plots of clinical outcome. CI = confidence interval, IA = ilioinguinal approach, MSA = modified Stoppa approach.

Clinical outcome was measured in 3 articles by the Matta modification of the Merle d’Aubigne score,^[22]^ with 65 patients in MSA group and 72 in the IA group. Excellent (17–18 points) and good results (15–16 points) were considered better clinical outcomes. This meta-analysis revealed no statistically significant differences between the 2 groups (*P* = .63; 95% CI: 0.35–1.87; Fig. 8).

#### 3.3.5 Complications

**Figure 9:**
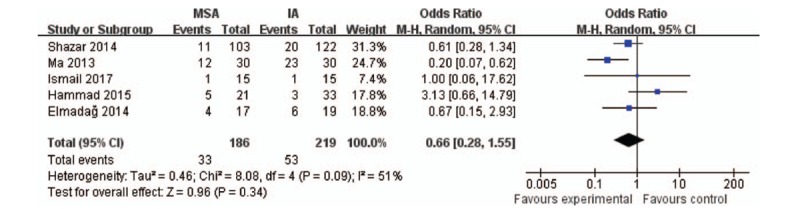
. Forest plots of complications. CI = confidence interval, IA = ilioinguinal approach, MSA = modified Stoppa approach.

**Figure 10:**
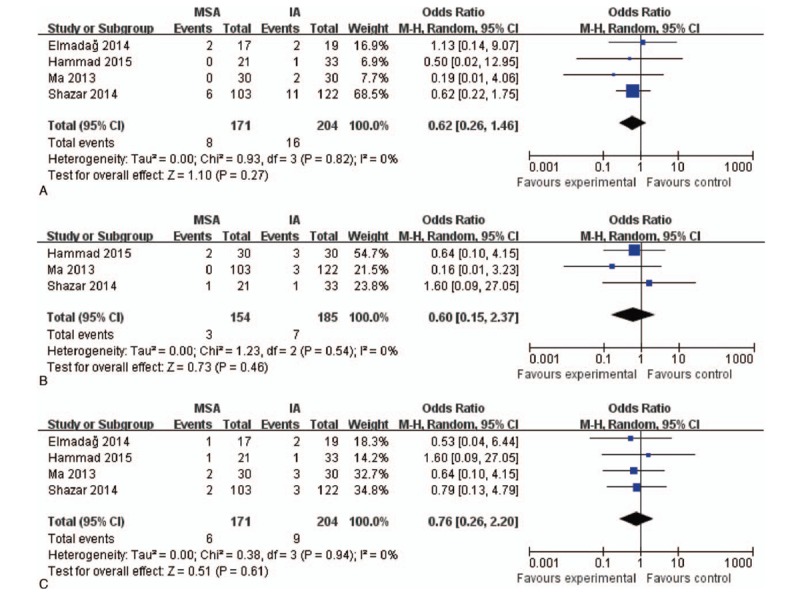
. Subgroup analysis of complications: A, infection; B, vascular injury; C, nerve injury. CI = confidence interval, IA = ilioinguinal approach, MSA = modified Stoppa approach.

All the included studies reported the complications between the 2 groups, with 186 patients in the MSA group and 219 in the IA group. This meta-analysis suggests no statistically significant difference between the MSA and IA groups (*P* = .34; 95% CI: 0.28–1.55; Fig. 9). In addition, we performed a subgroup analysis of complications, including infection, and vascular and nerve injuries. It also showed no statistically significant differences between the 2 groups (*P* = .62, *P* = .60, and *P* = .76, respectively; Fig. 10).

## 4 Discussion

On the basis of the currently available evidence, this meta-analysis suggests that MSA can significantly shorten operative time (*P* = .0002; 95% CI: −81.63 to −25.34), decrease intraoperative blood loss (*P* = .002; 95% CI: −446.11 to −97.07), and provide better reduction quality (*P* = .03; 95% CI: 1.08–3.39) as compared with IA in the treatment of anterior pelvic ring and acetabular fractures. In addition, in terms of clinical outcomes and complications, we found no significant differences between the 2 groups (*P* > .05).

IA has long been considered one of the most common approaches for the treatment of pelvic and acetabular fractures. It can be applied to almost all anterior pelvic ring and acetabular fractures, including anterior column fractures, anterior wall fractures, T-type fractures, anterior column with posterior and transverse fractures, and most double-column fractures. However, IA has been reported to be time consuming,^[14,15]^ easily increase intraoperative blood loss,^[14,15]^ and likely to cause nerve injury.^[2,3]^ MSA, as an alternative to IA for the treatment of anterior pelvic ring and acetabular fractures, has its own unique advantages such as protects the lateral femoral cutaneous nerve and femoral arteriovenous vessels and provides good visualization of the front and inner sides of pelvis and acetabular. Otherwise, because MSA needs to expose the corona mortis, the orthopedic surgeon is required to be fully familiar with the anatomy. Moreover, MSA also has some disadvantages such as obturator nerve injury,^[23]^ atrophy of rectus abdominis,^[14]^ and peritoneal perforation.^[5]^ Kim et al^[23]^ retrospectively analyzed the causes of obturator nerve injury caused by acetabular fractures with MSA. Obturator nerve injury was found to be related to the degree of quadrilateral plate displacement, especially when the displacement of the quadrilateral plate is >24 mm.

In this meta-analysis, we compared complication rates between MSA and IA groups in the treatment of anterior pelvic ring and acetabular fracture, and found no statistically significant differences between the 2 groups in terms of total complication rate, infection, and vascular and nerve injuries (*P* > .05). However, the total complication rate in IA group was 24.20%, which is significantly higher than that in the MSA group (17.74%). This may be caused by insufficient sample size and low quality of the included literature. Meanwhile, we assessed the reduction quality between the 2 groups. Anatomical and satisfactory outcomes were considered excellent qualities of reduction. We found that MSA had a significantly higher rate of reduction quality (84.46%) than IA (73.62%), but better reduction quality did not translate to better clinical results. No significant difference was found in clinical outcome between the 2 groups (*P* = .63). This may be related to the insufficient follow-up time.

Few scholars have conducted systematic reviews or meta-analysis to compare the efficacy between the 2 approaches in the treatment of anterior pelvic ring and acetabular fractures. To the best of our knowledge, only 1 article^[24]^ has reviewed and analyzed the difference between the 2 groups, which included 4 studies. Meena et al^[24]^ suggested that MSA can provide better reduction quality and lower operative time, which were verified in our research. However, in terms of complication rates, Meena et al suggested that MSA had a lower complication rate than IA, which was different from our results. This may be related to the number of articles included and the method of statistical calculations. We used a random-effects model to calculate the combined effect size to obtain a more conservative result.

This study has several limitations. First, only 5 studies with 405 patients were included in this meta-analysis and the sample sizes of the articles were not enough, which may be a potential source of bias. Second, the details of the operative techniques and preoperative combined injury in each patient were different. Third, although we searched the 3 most commonly used medical literature databases in strict accordance with the eligibility criteria, this meta-analysis included only 1 randomized controlled trial. Most of the included articles were retrospective studies, which may be a potential source of bias.

Therefore, more high-quality randomized controlled trials are needed to compare the clinical outcomes, radiographic outcomes, and complication rates between MSA and IA in the treatment of anterior pelvic ring and acetabular fractures. Meanwhile, studies with long-term follow-up periods should also be conducted.

## 5 Conclusion

This meta-analysis suggests that for anterior pelvic ring and acetabular fractures, MSA can significantly shorten the operative time, reduce the intraoperative blood loss, and provide better reduction quality than IA. In addition, in terms of clinical outcomes and complications, we found no significant differences between the 2 groups. High-quality randomized controlled trials with long-term follow-up are needed to verify our results.

## Author contributions

**Conceptualization:** Hao Wu.

**Data curation:** Hao Wu, LiJun Zhang, XiaoJun Jiang.

**Formal analysis:** Hao Wu, LiJun Zhang, XiaoJun Jiang.

**Investigation:** Hao Wu, LiJun Zhang, XiaoJun Jiang.

**Methodology:** XiaoJun Jiang.

**Project administration:** XiaoMin Guo.

**Resources:** XiaoMin Guo.

**Software:** XiaoMin Guo.

**Supervision:** XiaoJun Jiang.

**Validation:** XiaoMin Guo.

**Visualization:** LiJun Zhang.

**Writing – original draft:** Hao Wu, LiJun Zhang, XiaoJun Jiang.

**Writing – review & editing:** LiJun Zhang, XiaoJun Jiang.
